# *Aedes aegypti *uses RNA interference in defense against Sindbis virus infection

**DOI:** 10.1186/1471-2180-8-47

**Published:** 2008-03-17

**Authors:** Corey L Campbell, Kimberly M Keene, Douglas E Brackney, Ken E Olson, Carol D Blair, Jeffrey Wilusz, Brian D Foy

**Affiliations:** 1 Arthropod-borne Infectious Diseases Laboratory; Microbiology, Immunology, and Pathology Department, Colorado State University, Fort Collins, USA; 2 Laboratory Services Division, Colorado Department of Public Health and Environment, Denver, USA

## Abstract

**Background:**

RNA interference (RNAi) is an important anti-viral defense mechanism. The *Aedes aegypti *genome encodes RNAi component orthologs, however, most populations of this mosquito are readily infected by, and subsequently transmit flaviviruses and alphaviruses. The goal of this study was to use *Ae. aegypti *as a model system to determine how the mosquito's anti-viral RNAi pathway interacts with recombinant Sindbis virus (SINV; family *Togaviridae*, genus *Alphavirus*).

**Results:**

SINV (TR339-eGFP) (+) strand RNA, infectious virus titers and infection rates transiently increased in mosquitoes following dsRNA injection to cognate *Ago2*, *Dcr2*, or *TSN *mRNAs. Detection of SINV RNA-derived small RNAs at 2 and 7 days post-infection in non-silenced mosquitoes provided important confirmation of RNAi pathway activity. Two different recombinant SINV viruses (MRE16-eGFP and TR339-eGFP) with significant differences in infection kinetics were used to delineate vector/virus interactions in the midgut. We show virus-dependent effects on RNAi component transcript and protein levels during infection. Monitoring midgut *Ago2*, *Dcr2*, and *TSN *transcript levels during infection revealed that only *TSN *transcripts were significantly increased in midguts over blood-fed controls. Ago2 protein levels were depleted immediately following a non-infectious bloodmeal and varied during SINV infection in a virus-dependent manner.

**Conclusion:**

We show that silencing RNAi components in *Ae. aegypti *results in transient increases in SINV replication. Furthermore, *Ae. aegypti *RNAi is active during SINV infection as indicated by production of virus-specific siRNAs. Lastly, the RNAi response varies in a virus-dependent manner. These data define important features of RNAi anti-viral defense in *Ae. aegypti*.

## Background

*Aedes aegypti *is an important vector of arbovirus pathogens [1,2]. Understanding anti-viral defense mechanisms in mosquitoes will help define important features of vector competence and could lead to novel arbovirus control strategies. In contrast to what is known about mosquito responses to bacterial and fungal infections, little has been published concerning mosquito anti-viral defense (reviewed in [3]). In addition, there are fundamental differences between components of the canonical anti-viral defense mechanisms in vertebrates and invertebrates. For example, orthologous genes for the anti-viral effector molecules of vertebrates, such as type I interferons, protein kinase R, and 2'-5' oligoadenylate synthase, are not present in invertebrate genomes [3,4].

Signal transduction pathways known to be involved in bacterial and fungal innate immunity can be affected during arbovirus infections in vector mosquitoes and other insects (reviewed in [3]). For example, elements of the Toll signal transduction pathway are enriched in *Ae. aegypti *orally challenged with Sindbis virus (SINV, family, *Togaviridae; *genus *Alphavirus*) [5]. The Janus-kinase signal transducers and activators of transcription (Jak/STAT) and Toll signal transduction pathways also are activated in *Drosophila melanogaster *during infection with the insect pathogens Drosophila C virus (DCV; family, *Dicistroviridae*) and Drosophila X virus (DXV; family *Birnaviridae*) [6,7]. However, some key differences are evident between mosquitoes and the model organism *D. melanogaster*. For example, STAT activity is suppressed in *Aedes *cell culture during Flavivirus infection (an arbovirus) [8], but stimulated in *Drosophila *during DCV infection [6,8].

RNA interference (RNAi), an RNA silencing mechanism, is triggered by the recognition of intracellular long double-stranded RNAs (dsRNA) and is an important anti-viral response in invertebrates [9-12]. Because such evidence is lacking in mosquitoes, we relied on RNAi pathway functional information from model organisms, such as *Drosophila*. Researchers have long hypothesized that RNA silencing plays a role in anti-viral defense because of the presence of dsRNA structures formed by some RNA viruses in host cells [13]. Recent studies in mutant *Drosophila *adults confirmed the identity of specific genes that recognize, recruit and destroy viral RNAs [9-11,14]. Dicer-2 (Dcr2) is essential for initiation of anti-viral defense in *Drosophila*, as it processes long dsRNAs into small interfering RNAs (siRNAs) of about 21–23 bp [14,15]. Dcr2, with R2D2, loads one unwound strand of each siRNA into the multi-component RNA-induced Silencing Complex (RISC). In the effector phase, a RISC-loaded siRNA strand is used as a guide for target recognition and cleavage by Argonaute 2 (Ago2) [16]. In addition to Ago2 and other proteins, Tudor staphylococcal nuclease (TSN) is also a component of RISC [17]. This protein has multiple functions, including transcriptional co-activation, non-specific single-stranded RNA cleavage, and cleavage of hyper-edited dsRNA substrates [17,18]. Although it plays a clear role in RNAi, a link to anti-viral defense has not yet been shown. The *Ae. aegypti *genome has orthologs to all of these components [19,20].

Beginning in the mid-1990's, we provided evidence in both mosquito cell culture and adult mosquitoes that long dsRNA triggers RNA silencing, which suppresses replication of arboviruses [21-28]. More recently, we have shown that in *An gambiae*, *Ago2 *and *Ago3 *are required for defense against intrathoracically injected O'nyong-nyong virus (family, *Togaviridae; *genus *Alphavirus*) [29]. In addition, in a transgenic *Ae. aegypti *line, *Ago2 *is required for RNAi-mediated defense against Dengue virus (family *Flaviviridae*; genus *Flavivirus*) infection [19]. Arbovirus infection of vector mosquitoes is most influenced by midgut infection and escape barriers, and to a lesser extent, salivary gland infection barriers, which combine to influence the ability of a mosquito to serve as a competent vector (vector competence) [30-32]. A fuller understanding of the role of RNAi-mediated defense is needed, especially in light of the knowledge that *Ae. aegypti*, even with the anti-viral RNAi arsenal, is often unable to clear a virus infection before the virus disseminates to the salivary glands for transmission to a new host.

To determine whether RNAi components are required for *Ae. aegypti *anti-viral defense, recombinant Sindbis viruses (SINV) bearing enhanced green fluorescent protein (eGFP) markers were used. SINV is not naturally transmitted by *Ae. aegypti*, therefore, this comprised a model system with which to investigate the inherent features of anti-viral defense. *Dcr2*, *Ago2 *and *TSN *transcripts were silenced prior to oral infection by the recombinant SINV, TR339-eGFP. TR339, representing a Paleoarctic/Ethiopian SINV genotype, when fed at high titer, initiates modest *Ae. aegypti *midgut infections that are often cleared by 7 days, with consequent dissemination rates to distal tissues of about 40% [33,34]. Here, we show that silencing these RNAi components results in a transient increase in TR339-eGFP titers and infection rates, thus supporting roles for each of these genes in anti-viral defense. Importantly, we provide evidence of virus genome-derived small RNAs in un-manipulated SINV-infected mosquitoes, thus confirming RNAi activity.

In addition, we compared alterations in midgut RNAi component transcript and protein profiles during infection with two recombinant SINV strains that exhibit marked differences in growth kinetics in the mosquito. In contrast to TR339-eGFP, MRE16-eGFP, representing an Oriental/Australian SINV genotype, fed at high titer, efficiently and persistently infects *Ae. aegypti *midguts and disseminates to other tissues in about 80% of mosquitoes [35]. We found that both RNAi component transcript and protein levels varied in a virus-dependent manner, highlighting the need to consider various virus genotypes in understanding RNAi-virus interactions.

## Results and Discussion

### RNAi components in Ae aegypti

**Figure 1:**
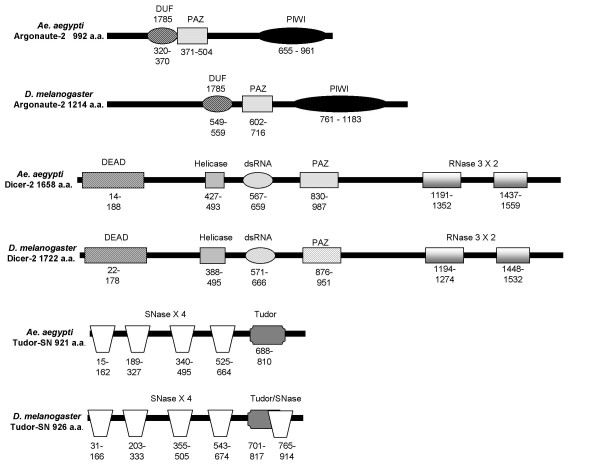
**Predicted protein domains of RNAi components used in this study compared to *Drosophila *orthologs**. *Ae aegypti *orthologs: *Ago2*, [Vectorbase: SUPP_AEDES003395], supercontig 1.89; *TSN*, [Vectorbase: AAEL000293], supercontig 1.5; *Dcr2*, [Genbank: AY713296], supercontig 1.221. *Drosophila *orthologs: *Ago2*, [Genbank: NP_648775], chromosome 3L; *Dcr2*, [Genbank: NP_523778], chromosome 2R; *TSN *[Genbank: NP_612021], chromosome 3L. "dsRNA", dsRNA binding; "DUF", domain of unknown function; "DEAD", helicase domain; "PAZ", small RNA binding domain; "PIWI", double-stranded RNA guided RNA cleavage; "RNase", RNA nuclease; "SNase", staphylococcal nuclease; "Tudor", domain of unknown function [51].

*Ae. aegypti Ago2*, *Dcr2*, and *TSN *genes were identified by homology to *D. melanogaster *and the *An. gambiae *orthologs. These genes are present as single copy 1:1:1 orthologous trios. *Ae. aegypti *orthologs encode conserved protein domains that identify them as RNAi components (Figure 1). The major catalytic enzyme, *Ae aegypti *Ago2, shares 42.7% amino acid (a.a.) identity with the *An. gambiae *ortholog and 33.2% identity with the *D. melanogaster *ortholog (Additional File 1A). Similarly, *Ae. aegypti *Dcr2 shares 51.3% a.a. identity with *An. gambiae *Dcr2 and 31.1% with *D. melanogaster *(Additional File 1B).*Ae aegypti *TSN shows more conservation across species, with 69.7% and 63% a.a. identity, respectively (Additional File 1C). These levels of similarity are common among proteins of these species [20].

### Effects of Ago2, Dcr2, or TSN dsRNA injection on TR339-eGFP infection

**Figure 2:**
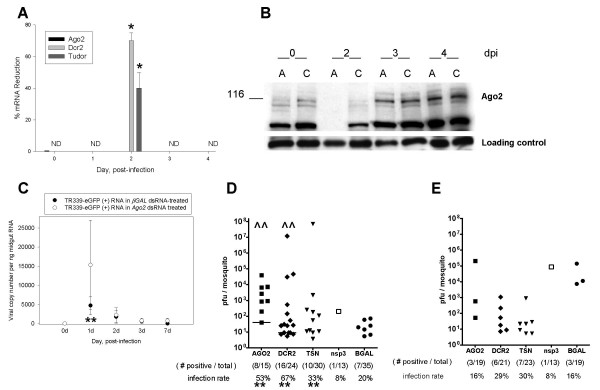
**TR339-eGFP virus infection transiently increased in mosquitoes receiving a Ago2, Dcr2, or TSN dsRNA injection**. (A) qRT-PCR analysis. Percent reduction in cognate *Dcr2*, and *TSN *mRNA levels, relative to actin, in midguts following *Ago2*, *Dcr2 *or *TSN *dsRNA treatment. "ND", none detected. Asterisks indicate statistical significance (Mann Whitney U test P ≤ 0.05). Error bars indicate standard error of three experimental replicates. (B) Immunoblot. Evidence of Ago2 protein silencing following dsRNA injection. "A" indicates *Ago2 *dsRNA-injected; "C" indicates *β*GAL injected controls. "0" days post-infection corresponds to female midguts harvested prior to virus meal. In each lane, equivalent amounts of protein extract from a pool of six midguts were separated by SDS-PAGE, blotted and probed with anti-Ago2 antibody. The loading control is a 19 kDa anti-Ago-2 cross-reacting band. Blot shown is from a single experiment and is representative of three independent replicates. (C) Two-step qRT- PCR showed that TR339 positive strand (+) RNA significantly increased in midguts of *Ago2 *dsRNA-injected mosquitoes over *βGAL *dsRNA controls at 1 dpi (P ≤ 0.05, Mann-Whitney U test). Pools of five midguts per group were used. Error bars depict standard error of three independent feedings. Viral (+) strand RNA copy numbers were calculated using the standard curve method. Asterisk indicates statistical significance, Mann-Whitney U test p ≤ 0.05. (D) TR339-eGFP viral titers of individuals at 4 dpi in *Ago2, Dcr2*, and *TSN *dsRNA-injected mosquitoes, with *βGAL *dsRNA controls. "**" indicates infection rate significantly higher than *βGAL *controls. "^^" above the graph indicates viral titers significantly higher than *βGAL *controls. Closed squares, Ago2 dsRNA; closed circles, *βGAL *dsRNA; closed diamonds, *DCR2 *dsRNA; closed triangles, *TSN *dsRNA; open squares, *nsP3*. Horizontal line indicates the median titer; no line indicates a median value of zero. "()" indicates (number of mosquitoes in each group per number positive). (E) TR339-eGFP viral titers at 7 dpi.

To determine whether *Ae. aegypti *RNAi components are required for defense against SINV TR339-eGFP, *Dcr2*, *Ago2*, and *TSN *were silenced by injection of dsRNA 3 days prior to an infectious bloodmeal. We predicted that dsRNA injection would transiently silence expression of these genes, because pre-existing RISC proteins should be available to initiate the silencing process. Although both *TSN *and *Dcr2 *transcripts showed evidence of transient silencing in midguts following dsRNA injection, there was no detectable reduction in *Ago2 *mRNA levels by qRT-PCR (Figure 2A). However, there was a reduction in Ago2 116 kDa protein levels, compared to non-specific dsRNA-injected controls, in unfed midguts just prior to an infectious bloodmeal and at 2 dpi (Figure 2B). The silencing was no longer evident by 3 dpi. On average, Ago2 protein levels were reduced to 49% of controls (+/- 16%) immediately prior to the virus meal and 22% of controls (+/- 17%) at 2 dpi. Previous reports showed that dsRNA effectively silences most targeted genes in the midgut of several different mosquito species for >7 days post-injection [19,36,37]. In the current study, silencing of RNAi components is more transient.

To determine if *Ago2 *dsRNA-injections altered virus replication, viral (+) and (-) RNA strands were detected by two-step qRT-PCR of TR339-eGFP-infected mosquito midgut pools. TR339-eGFP (+) strand RNAs showed a transient increase over beta-galactosidase (β*GAL*)-injected controls at 1 dpi (Figure 2C). The transient increase showed a high variance, probably due to variability in the absolute number of ingested viral particles per mosquito. Importantly, although viral copy number varied from experiment to experiment, in each replicate, *Ago2 *dsRNA-treated mosquito midguts showed higher viral copy number than *bGAL*-treated. In contrast, no changes were observed in the copy number of TR339-eGFP (-) strand RNA (data not shown).

With the knowledge that RNAi component silencing requires a functional RISC, we predicted that enhancement of viral titers would be transient. Indeed, *Ago2 *silencing resulted in transient increases in TR339-eGFP infection rates in whole mosquitoes (*χ*^2 ^= 5.6, P = 0.02) and increased median viral titers (Mann-Whitney U test, P = 2e-15) (Figure 2D, Table 1). Viral eGFP fluorescence in midguts of *Ago2 *-silenced mosquitoes was higher than controls at 4 dpi (*χ*^2 ^= 16.9, p = 4e-5); however, enhanced viral infection and replication were no longer evident by 7 dpi (Figure 2E; Table 1). Together, these results indicate the requirement for *Ago2 *in anti-viral defense during TR339-eGFP infection.

**Table 1:** TR339-eGFP Infection Profiles following dsRNA Injection. Significantly enhanced viral eGFP fluorescence occurs at 4 dpi in midguts following *Ago2 *dsRNA treatment and in disseminated tissues at 7 dpi following *TSN *dsRNA treatment.

		Day 4		Day 7	
		
dsRNA		Midgut	Dissemination	Midgut	Dissemination
*βgal*	Light	28/47 (59.6%)		38/76 (50%)	
	Moderate	2/47 (4.3%)		5/76 (6.6%)	
	Heavy	3/47 (6.4%)		5/76 (6.6%)	
	Total	33/47 (70.2%)	10/33 (30%)	48/76 (63.2%)	12/48 (25%)
*Ago2*	Light	7/25 (28%)		20/53 (37.7%)	
	Moderate	6/25 (24%)		14/53 (26.4%)	
	Heavy	12/25 (48%)		5/53 (9.4%)	
	Total	25/25 (100%)**	12/25 (48%)	39/53 (73.5%)	5/39 (12.8%)
*Dcr2*	Light	9/20 (45%)		7/19 (36.8%)	
	Moderate	2/20 (10%)		2/19 (10.5%)	
	Heavy	0/20 (0%)		0/19 (0%)	
	Total	11/20 (55%)	5/11 (45.5%)	9/19 (47.3%)	3/9 (33.3%)
*TSN*	Light	12/31 (38.7%)		10/37 (27%)	
	Moderate	4/31 (12.9%)		2/37 (5.4%)	
	Heavy	0/31 (0%)		3/37 (8.1%)	
	Total	16/31 (51.6%)	5/16 (31.3%)	15/37 (40.5%)^^	15/15 (100%)**
*nsP3*	Light	1/32 (3.1%)		1/28 (3.6%)	
	Moderate	0/1 (0%)		0/28 (0%)	
	Heavy	0/32 (0%)		0/28 (0%)	
	Total	1/32 (3.1%)^^	0/1 (0%)	1/28 (3.6%)^^	0/1 (0%)

"()" indicates percentage eGFP detected in each group.

"**" indicates infection rate higher than *βGAL *controls. "^^" indicates infectious rate significantly lower than *βGAL *controls.

Light: 1–33% of the midgut infected; Moderate: 34–66%; Heavy: 67–100%; illustrations of infection patterns have been previously reported [33, 35].

A similar pattern of enhanced viral infection was observed following *Dcr2 *dsRNA treatment. Transient *Dcr2 *silencing resulted in temporary increases in TR339-eGFP infection rates compared to controls (*χ*^2 ^= 13.0, P = 0.0003) (Figure 2D), however increases in eGFP fluorescence were not significant (Table 1). At 4 dpi, the *Dcr2 *dsRNA-injected group also had significantly higher median titers than β*GAL *controls (Figure 2D) (Mann Whitney U test, P = 0.0001), and, by 7 dpi, the effect was abrogated (Figure 2E). As was seen with Ago2, there is a clear requirement for Dcr2 during anti-viral defense.

*TSN *silencing showed less striking enhancement of viral infection than either *Ago2 *or *Dcr2*. At 4 dpi, median viral titers of *TSN*-silenced mosquitoes were not significantly different from controls (Figure 2D); however infection rates were slightly higher (Mann Whitney U test, P = 0.03). By 7 dpi, the infection pattern, detected by eGFP fluorescence showed significantly higher dissemination (*χ*^2 ^= 5.2, p = 0.02), even though clearing of midgut infection was evident (Table 1). This fluorescence was primarily present in hemolymph with very little in peripheral tissues. Concurrently, viral titers were reduced to levels below those of controls (Figure 2E). Importantly, detection of eGFP in this system is indicative of viral protein production. Therefore, the eGFP fluorescence in hemocytes coupled with reduced viral titers indicates that only a small amount of live virus escaped the midgut. Alternatively, hemocytes became infected in the midgut and then migrated to other tissues. Nevertheless, the virus was limited in its ability to replicate further. All of the results suggest that the effects of silencing wane by 7 dpi. This could be due to the decay of exogenously-administered dsRNA, or transient stimulation of infection rates at 4 dpi might be overcome by a stimulated anti-viral defense pathway by 7 dpi.

SINV non-structural protein 3 (nsP3) participates in replication complex formation [38,39]. Injection of dsRNA derived from TR339 nsP3 showed that this treatment nearly ablated viral replication in 12 of 13 mosquitoes at both 4 and 7 dpi (Figure 2D, 2E, Table 1). Midgut eGFP fluorescence was significantly reduced in nsP3-targeted mosquitoes (*χ*^2 ^= 8.2, P = .00042) (Table 1). This evidence demonstrates the ability of virus RNA-derived dsRNA to trigger anti-viral RNAi in *Ae aegypti*.

### Transient *Ago2*, *Dcr2 *or *TSN *silencing does not increase mortality during TR339-eGFP infection

Mutation of genes encoding RNAi pathway components in *D. melanogaster *resulted in increased mortality of flies infected with invertebrate viral pathogens [9,14]. In addition, there is evidence that some arboviruses cause pathology in infected mosquito tissues [40-42]. To determine whether RNAi components prevent mortality during SINV infection, we examined survival curves of TR339-eGFP infected *Ae. aegypti *injected with *Ago2, Dcr2 *or *TSN *dsRNA. Results indicated that mortality was not increased over *βGal *dsRNA-injected controls (Additional File 2). The transient effects of the dsRNA injection or the weak replication of TR339-eGFP may have been factors in the lack of mortality. Therefore, the mortality study was repeated with *Dcr2 *silenced mosquitoes infected with MRE16-eGFP. Again, there was no significant enhancement of mosquito mortality (Additional File 3). Nevertheless, to confirm these preliminary findings, a gene knock-out system for mosquitoes would be required.

### Evidence of RNAi pathway activity

**Figure 3:**
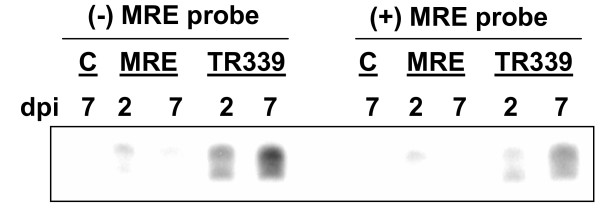
**Small viral RNAs are detected**. Small RNAs (18 to 25 nts) were isolated from SINV-infected mosquitoes at 2 or 7 days post-infection, size-selected by gel electrophoresis and hybridized to pools of strand-specific probes representing the complete viral genome indicated. Eluted products were separated on a 5% acrylamide gel and detected by autoradiography. "Dpi", day, post-infection, "MRE", MRE16-eGFP-infected, "TR339", TR339-eGFP-infected, "B", bloodfed control.

**Figure 4:**
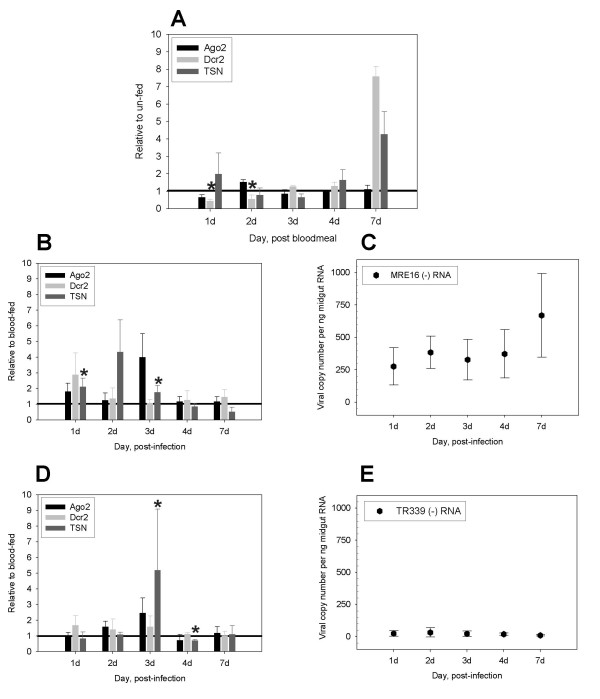
**Non-infectious bloodmeal depletes *Dcr2 *transcripts; TSN transcript levels are enriched in mosquito midguts during SINV infection**. (A) Effects of bloodfeeding on *Ago2, Dcr2*, and *TSN *transcript levels. Relative midgut transcript levels from bloodfed mosquitoes were normalized to unfed controls. Transcript levels at 1.0 indicate no change over unfed control levels. (B) Effects of MRE16-eGFP infection on midgut transcript levels relative to un-infected bloodfed controls. (C) MRE16-eGFP (-) strand RNA detected in midgut total RNA from panel (B). (D) Effects of TR339-eGFP infection on midgut transcript levels relative to un-infected bloodfed controls. (E) TR339-eGFP (-) strand RNA levels in midgut total RNA from panel (D). Mosquitoes were fed either a bloodmeal or the virus indicated. (A, B, D) Total RNA from pools of 5 midguts were used for qRT-PCR of *Dcr2*, *Ago2*, *TSN *and *Act1 *transcripts. Changes in relative transcript levels of each RNAi component were determined by using actin as an internal reference standard and normalizing each value to that of the control group indicated. Relative expression levels were determined using the comparative Ct method; transcript levels at 1.0 indicate no change over bloodfed control levels. Significant changes in expression levels are shown by asterisks (p ≤ 0.05, Mann-Whitney U test). Error bars depict standard error of three independent feedings. (C, E) Viral transcript copy numbers per nanogram midgut RNA were determined by two-step quantitative RT-PCR; these were calculated using the standard curve method. Error bars indicate standard error of three independent virus infections.

The production of sequence-specific small RNAs is a hallmark of RNAi defense. RNAi activity was confirmed during natural TR339-eGFP and MRE16-eGFP infections by identifying viral small RNAs in adult female mosquitoes (Figure 3). We used MRE16-eGFP whole genome capture probes, as these two viruses share about 75% nucleotide identity [43]. TR339-eGFP infected mosquitoes accumulated far more small viral RNAs between 2 and 7 dpi than MRE16-eGFP infected mosquitoes. Small viral RNAs from MRE16-eGFP were barely detectable only at 2 dpi. These data are interesting in light of the differences in replication kinetics of these two viruses (Figure 4). They could indicate a correlation between replication efficiency in the mosquito and susceptibility to the anti-viral RNAi defense, or they could point to a viral suppressor of RNAi in the MRE16-eGFP virus [4,10,44]. It is also apparent that TR339-eGFP-derived (+) sense small RNAs are more abundant than (-) sense small RNA in mosquito tissues. This suggests that the (+) virus strand is targeted by RNAi more efficiently than the (-) virus strand or that diced (+) sense viral RNA is preferentially stabilized in the RISC and used as the guide strand. It could also suggest that the primary viral dsRNA trigger is (+) sense viral RNA secondary structure, rather than (+) and (-) dsRNA of the replicative intermediate.

### Mosquito and viral transcript analysis

The mosquito midgut is the initial site of arbovirus replication and thus is suspected to be the first site of anti-viral defense. Blood-feeding alone results in reduced midgut *Dcr2 *mRNA levels at 1 and 2 days post-feeding (Figure 4A) (Mann-Whitney U Test, P = 0.02 and P = 0.02, respectively). Although fluctuations were also observed in *Ago2*, *Dcr2 *and *TSN *mRNA levels at other timepoints, they were not significantly different from controls.

TR339-eGFP and MRE16-eGFP replicate and amplify in the midgut for 2 to 3 days prior to escape to other tissues [33,35]. Mosquitoes were orally infected with either MRE16-eGFP or TR339-eGFP, and RNAi component midgut transcript levels were determined relative to un-infected blood-fed controls (Figure 4B, 4D; Additional File 4). The mosquito's overall transcript pattern differed for each virus strain. During MRE16-eGFP infection, *TSN *transcript levels, but not *Ago2 *or *Dcr2*, were significantly enriched over controls at 1 and 3 dpi (Mann-Whitney U Test, P = 0.05 and P = 0.04, respectively), and highly variable at 2 dpi. In contrast, during TR339-eGFP infection, *TSN *transcript levels were not significantly enriched until 3 dpi, when a 5-fold enrichment was followed by a sharp decrease at 4 dpi (Mann-Whitney U Test, P = 0.04 and P = 0.04, respectively).

Enhancement of *Ae. aegypti TSN *transcripts was previously reported in microarray analyses of SINV MRE16 infection [5]. The *TSN *transcript enrichment in *Ae. aegypti *during SINV infection contrasts with reports of *TSN *depletion in *Drosophila *during DCV infection [6]. DCV has a single-stranded positive sense RNA genome, as does SINV. However, DCV, a picorna-like virus, is an insect-specific pathogen whereas SINV is an arbovirus, and by definition, infects both mosquitoes and vertebrates and establishes persistent, non-lethal infections in the insect vector.

Detection of (-) strand RNA served as evidence of viral replication in the samples used for RNAi component transcript analysis. Interestingly, viral replication markedly differed between the two SINV strains. MRE16-eGFP (-) RNA levels increased over the 7 day period, from an average of 276 copies per nanogram (ng) midgut RNA at 1 dpi to 670 copies at 7 dpi (Figure 4C). In contrast, TR339-eGFP (-) RNA was undetectable in 2 of 3 experimental replicates at 1 dpi and increased to an average high of 33 copies at 2 dpi. Over the remainder of the 7 day time course, (-) strand RNA levels remained low (Figure 4E). The steady increase in (-) strand SINV RNA in mosquitoes over the 7 day period differs from that observed in vertebrate cell culture. In vertebrate cells, (-) strand RNA synthesis ceases about 30 hr post-infection, due to the cleavage of nsP1-4 products shifting replication towards (+) strand synthesis [45]. The continued increase in (-) strand RNA in midguts in contrast to that of vertebrate cell culture is probably due to the progressive cell-to-cell spread of virus through the tissue, whereas, in vitro cell culture infection is initiated by bathing all cells in virus.

Importantly, despite clear differences in MRE16-eGFP and TR339-eGFP growth characteristics, no statistically significant changes from controls were seen for *Ago2 *or *Dcr2 *mRNAs during SINV infection (Figure 4B, 4D). Further, enrichment of *TSN *transcripts occurred at different times following infection for each virus strain. Transcript enrichment occurred early during MRE16-eGFP infection concomitant with viral replication, whereas, *TSN *enrichment in TR339-eGFP infected midguts was delayed, possibly because viral replication remained at low levels. These results suggest that TSN may act as a sensor for the RNAi pathway.

### Ago2 protein profiles

**Figure 5:**
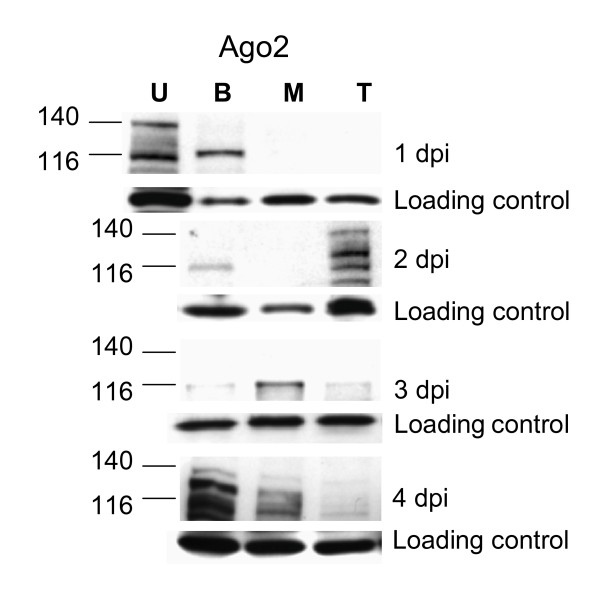
***Ae aegypti *Ago2 protein depletion and accumulation varied in a virus-dependent manner**. Protein was extracted from pools of ten midguts from unfed, bloodfed or virus/bloodfed adult female *Ae aegypti *at the time points indicated. Equivalent protein amounts were separated by 4–15% gradient PAGE prior to blotting. Anti-*Ae aegypti *Ago2 antibody recognizes the carboxy-terminal peptide sequence YERMQIRTEIQDGHPMFFV. The loading control is a 19 kDa Ago2 cross-reacting band. "U", unfed female, "B", non-infectious bloodfed female, "M", MRE16-eGFP-infected, "T", TR339-eGFP-infected. Blots are representative of two independent experiments.

The lack of statistically significant increases in *Ago2 *transcript levels during SINV infection led us to ask whether protein levels fluctuated. Figure 5 shows Ago2 protein profiles in MRE16-eGFP and TR339-eGFP infected midguts compared to unfed and un-infected blood-fed controls. Bloodfeeding alone reduces Ago2 protein levels compared to midguts from unfed mosquitoes, and these proteins re-accumulated over a time period concomitant with the expected time course of bloodmeal digestion, vitellogenesis, and a return to the unfed state.

Virus infection influenced Ago2 protein patterns in the midgut. Depletion of Ago2 protein occurred during either TR339-eGFP or MRE16-eGFP infection at 1 dpi; thereafter, Ago2 protein levels varied in a virus-dependent manner, and were distinct from the profile seen in un-infected controls. In MRE16-eGFP-infected midguts, Ago2 protein remained depleted at 2 dpi and increased over un-infected controls at 3 dpi. This enrichment corresponds to the concurrent, though statistically insignificant, increase in *Ago2 *transcripts (Figure 4B). In contrast, during TR339-eGFP infection, Ago2 protein levels were enriched and evident as multiple bands at 2 dpi, were roughly equivalent to bloodfed controls at 3 dpi, and reduced again at 4 dpi. By 7 dpi, protein profiles for all virus-infected midguts were similar to those of bloodfed controls (data not shown). When comparing natural fluctuations of Ago2 protein levels in Figure 4 with those from dsRNA-injected mosquitoes in Figure 2, it is apparent that injection of non-specific dsRNA results in depletion of Ago2 levels.

### Summary

In summary, the low level of TR339-eGFP replication in midguts, coupled with a lack of Ago2 protein depletion and the detection of small viral RNAs, indicates that anti-viral RNAi defense is active during infection with this virus. The low steady state levels of (-) strand RNA detected by qRT-PCR do not reveal fluctuations that could occur due to RNA replication or degradation. Therefore, further analysis is required to determine whether these low levels are due to inefficient replication or an active RNAi response. A much different pattern was observed during MRE16-eGFP infection. MRE16-eGFP replicates to significantly higher copy number than TR339-eGFP while somehow preventing the accumulation of small viral RNAs indicative of effective RNAi defense. The specifics of this evasion or suppression mechanism are not understood and warrant further study. Importantly, these results underscore the different effects that can occur with different arboviruses and indicate that specific effects on the RNAi pathway need to be characterized separately for each virus system.

## Conclusion

The dissimilar growth kinetics of the two recombinant SINV strains might be due to differences in ability to evade RNAi defense. The parallel detection of virus replication, viral small RNAs, and RNAi component transcripts and proteins indicates that anti-viral defense occurs early in infection. These data support the hypothesis that RNAi anti-viral defense occurs in *Ae. aegypti *against SINV infection. The reduction of Ago2 protein levels soon after non-infectious blood or SINV/blood meals suggests that expression of new defense proteins may be a rate-limiting step in anti-viral defense. Variations in *Ago2*, *Dcr2*, and *TSN *transcripts and protein levels, as well as variation in accumulation of viral small RNAs from infected mosquitoes, suggests that anti-viral defense activity differs in a virus-dependent manner, potentially because some viruses have the ability to avoid or suppress the RNAi response. Our data also suggest that regulation of the RNAi pathway in mosquitoes, as well as RNAi pathway/arbovirus interactions, differ from those in non-vector insects infected with pathogenic viruses. The first evidence for this is that *TSN*, a transcriptional co-activator, transcripts are enriched during arbovirus infection, whereas, in *Drosophila*, this does not occur [6]. Moreover, mosquitoes with transiently silenced RNAi components, harbouring higher SINV titers, did not exhibit increased mortality as compared to controls, unlike *Drosophila *infected with pathogenic insect viruses.

### Future Directions

The differential effects of MRE16-eGFP and TR339-eGFP on RNAi pathway activity indicate a need to investigate the viral features that prevent or evoke RNAi pathway activity. A deeper understanding of these virus-specific effects will be important to defining the characteristics of an efficient arbovirus and the limitations of RNAi anti-viral defense in arbovirus vectors.

## Methods

### Virus infections and titrations

MRE16-eGFP and TR339-eGFP virus stocks were generated from SINV infectious cDNA clones with inserts of enhanced GFP (eGFP) driven by the second sub-genomic promoter, using standard methods [35,46,47]. MRE16-eGFP was passed once in baby hamster kidney (BHK-21) cells and twice in *Aedes albopictus *clone C6/36 cells. TR339 5'2J-eGFP was passed once in BHK-21 cells and three times in C6/36 cells.

For mosquito feedings, all viruses were diluted as indicated in de-fibrinated sheep blood and provided at 37°C in a water jacketed artificial feeder with parafilm membrane. An aliquot of each SINV stock was titered by viral plaque assay on Vero cells and the other aliquots were stored at -80°C and diluted prior to feeding. TR339-eGFP blood-meals contained approximately 3.3 × 10^8 ^pfu/ml and MRE16 meals contained 2.2 × 10^7 ^pfu/ml. Representative mosquitoes from each group were selected for dissection and eGFP detection with an Olympus epi-fluorescence microscope to confirm midgut infection with each batch of virus. Viral titers from individual mosquitoes were determined by plaque assays of filtered mosquito homogenates as previously described [29].

### Mosquitoes and intrathoracic injection of dsRNA

Colonized *Ae. aegypti *Rexville D- Higgs' White Eye (HWE) mosquitoes were reared at 28°C, 80% relative humidity, with a photoperiod of 14:10 (L:D). Adults were provided with a sugar source and water and held in the same conditions during the extrinsic incubation period.

dsRNA was transcribed from templates generated from cDNA clones following PCR amplification with 5' primer extensions bearing a T7 RNA polymerase promoter (Additional File 4) and the protocol described with the Megascript dsRNA kit (Ambion, Austin, TX). dsRNA was purified with phenol/chloroform extraction and diluted to a concentration of 1.0 μg/μl in phosphate-buffered saline (PBS). Adult female *Ae. aegypti *(2–3 days post emergence) were anesthetized with cold, held on ice and intrathoracically injected with approximately 500 nanograms dsRNA. Three days later, mosquitoes were harvested for protein extraction or fed a blood-meal containing 8 log pfu/ml TR339-eGFP.

### Visualization of TR339-eGFP infection in *Ae. aegypti *mosquitoes

Evidence of virus infection and dissemination was observed at 4 and 7 days post-infection (dpi) by dissecting mosquitoes in PBS and observing eGFP fluorescence of midguts and peripheral tissues by epi-fluorescence microscopy (Olympus, Center Valley, PA).

### Quantitative reverse transcriptase PCR (qRT-PCR)

Pools of 5 midguts from three independent feedings were frozen at -80C prior to extraction of total RNA using Trizol Reagent (Invitrogen, Carlsbad, CA). Genomic DNA was removed using TurboDNase I (Ambion, Austin, TX) in a 100 μl reaction for 1 hour at 37°C and inactivated using DNase I inactivation beads. Samples were stored as ethanol slurries at -80°C in multiple aliquots. Amplicons were prepared in an Opticon 2 real-time PCR thermocycler (BioRad, Hercules, CA) in triplicate using SYBR Green One step qRT-PCR reagents (Invitrogen, Carlsbad, CA) in 20 μl reactions with 2.0 ng total RNA per well. Additional File 4 shows the qPCR primers used. Cycling parameters followed the manufacturer's recommendations and used a 57°C extension temperature. Relative transcript levels were determined using the comparative Ct analytical method with an actin reference standard and the blood-fed control as calibrator [48].

### Viral RNA qRT-PCR

Two step amplification of (-) strand SINV RNA was performed according to the methods of Richardson [49]. Briefly, 10 ng mosquito midgut total RNA were used for reverse transcription reactions. For MRE16-eGFP and TR339-eGFP, the forward primer (Additional File 4) was used to transcribe (-) strand RNA. A 2 μl aliquot from the reverse transcriptase reaction was used in quantitative real-time PCR using ABI Powerscript (Applied Biosystems, Foster City, CA). Viral copies were calculated using the standard curve method [49].

### Immunoblot analyses

All midguts were harvested into PBS with protease inhibitor cocktail (P2714, Sigma, St Louis, MO), flash frozen in dry ice, stored at -80C, and processed simultaneously for immunoblot analysis. Pools of 10 adult female midguts were triturated in 1% sodium dodecyl sulfate, protease inhibitor cocktail, 20 micrograms/ml phenylmethylsulfonylfluoride (Sigma-Aldrich, St Louis, MO) diluted in PBS. Equivalent amounts of protein extracts (10 υg) were separated using standard denaturing conditions on a 4 to 15% gradient gel and transferred to PVDF membrane using a tank blotter (BioRad, Hercules, CA) and transfer buffer containing 25 mM Tris, pH 8.3, 192 mM glycine. Ago2 was detected using a 1:100 dilution of affinity purified rabbit anti- *Ae. aegypti *Ago2 antibody recognizing the peptide sequence C-YERMQIRTEIQDGHPMFFV (QCB, Hopkinton, MA). Antibody affinity purification followed methods recommended by the antibody service company (QCB, Hopkinton, MA). The corresponding genetic code is present only once in the *Ae. aegypti *genome [50]. Multiple banding patterns were observed; the predicted molecular weight of Ago2 is 115 kilodaltons (kDa). The loading control was a 19 kDa anti-Ago2 antibody cross-reacting band that remained constant during all conditions. The secondary antibody was goat anti-rabbit IgG conjugated to horseradish peroxidase (Abcam, Cambridge, MA). Proteins were detected using ECL Plus (GE Healthcare, Piscataway, NJ) detection reagents and ECL Hyperfilm (GE Healthcare, Piscataway, NJ).

### Small RNA detection

Mosquitoes were infected as described or fed blood alone and held for 2 or 7 days prior to storage in Trizol reagent (Invitrogen, Carlsbad, CA) at -80°C. Total RNA extraction followed manufacturer's recommendations. Small RNAs corresponding to 18–25 nucleotides were separated on a 7 M urea, 12% acrylamide gel, then excised and eluted. These small RNAs were treated with calf intestinal alkaline phosphatase to remove 5' phosphates and radiolabeled using T4 polynucleotide kinase and gamma-^32^P ATP. Radiolabeled RNAs were mixed with pooled biotinylated viral probes, described below, in 10 mM Tris, pH 7.6 and denatured for 1 min at 95°C. Reaction mixtures were adjusted to 400 mM NaCl and incubated at 42°C for 4 hr to allow hybridization. Streptavidin agarose beads were added to the mixture, incubated at room temperature for 10 min to allow capture of the biotinylated probes, and washed 4 times in 10 mM Tris, pH 7.6, 400 mM NaCl. Radiolabeled small RNAs were eluted from the probes in 10 mM Tris, pH 7.6 at 95°C for 5 min. Eluted small RNAs were concentrated by ethanol precipitation, separated on a 5% acrylamide gel containing 7 M urea, and detected on a Typhoon phosphorimager (GE Biosciences).

Biotinylated, strand-specific, viral RNA hybrid selection probes were prepared by restriction endonuclease digestion of the infectious MRE16-eGFP cDNA clone plasmid, followed by PCR amplification of 3 to 5 kB portions of the viral genome (Additional File 4). Each of these was used as a template in transcription of biotinylated probes of (-) or (+) strand polarity. Probes representing the (-) or (+) viral strands were equally pooled to provide genome- or anti-genome-wide coverage in the hybridization step. TR339-eGFP was not used as a hybridization probe because of inefficiencies in biotinylated RNA probe.

## Authors' contributions

CLC contributed to project design, did the majority of the experiments and wrote the manuscript. DEB determined viral copy number. KMK did the majority of the *Ago2 *dsRNA experiments. JW performed the small viral RNA detection. CDB and KEO contributed to the design of the project and edited the manuscript. BDF conceived the project, performed virus feeding experiments and edited the manuscript. All authors read and approved the final manuscript.

## Supplementary Material

Additional file 1 **Amino acid conservation of Ago2, Dcr2, and TSN across species**. Sequence alignment. Click here for file

Additional file 2 **Ago2, Dcr2, or TSN silencing does not increase mosquito mortality during TR339-eGFP infection**. Life Table analysis. Click here for file

Additional file 3 **Dcr2 silencing does not increase mosquito mortality during MRE16-eGFP infection**. Life Table analysis. Click here for file

Additional file 4 **Primer Table**. PCR primer sequences. Click here for file
