# Supplementary material for: Aedes aegypti uses RNA interference in defense against Sindbis virus infection
Source: BMC Microbiol. 2008 Mar 17;8:47. doi: 10.1186/1471-2180-8-47 (PMC2278134; doi:10.1186/1471-2180-8-47)
Supplement: Additional file 1 — Amino acid conservation of Ago2, Dcr2, and TSN across species. Sequence alignment. [file 1471-2180-8-47-S1.pdf]

Campbell and others, BMC Microbiology

Additional file 1A

```
                         1                                                50
   Ago2.aae      (1) --------------------------------------------------
  Ago2.agam      (1) --------------------------------------------------
   Ago2b.dm      (1) MGKKDKNKKGGQDSAAAPQPQQQQKQQQQRQQQPQQLQQPQQLQQPQQLQ

                         51                                              100
   Ago2.aae      (1) --------------------------------------------------
  Ago2.agam      (1) --------------------------------------------------
   Ago2b.dm     (51) QPQQQQQQQPHQQQQQSSRQQPSTSSGGSRASGFQQGGQQQKSQDAEGWT

                         101                                             150
   Ago2.aae      (1) --------------------------------------------------
  Ago2.agam      (1) --------------------------------------------------
   Ago2b.dm    (101) AQKKQGKQQVQGWTKQGQQGGHQQGRQGQDGGYQQRPPGQQQGGHQQGRQ

                         151                                             200
   Ago2.aae      (1) ------MILNARYLYIPCRQKQQ------QQQQQPQQQQQHQQKQQSQQQ
  Ago2.agam      (1) ------FLAQLGKRYQGHYSNNS------HRNNNHNHNHNHNNSSSLNNG
   Ago2b.dm    (151) GQEGGYQQRPPGQQQGGHQQGRQGQEGGYQQRPSGQQQGGHQQGRQGQEG

                         201                                             250
   Ago2.aae     (39) QQQQQQQQRSKEQGSQ----------QQQRPQQQAQQQQPSQQQQQSQKQ
  Ago2.agam     (39) RNSSSSHSLNKGHSSSSHKVHCSRDPRNSKGMHRSSNSRRAHHNSSSSRG
   Ago2b.dm    (201) GYQQRPPGQQQGGHQQGRQGQEGGYQQRPSGQQQGGHQQGRQGQEGGYQQ

                         251                                             300
   Ago2.aae     (79) QHPQQQQQQRPQKQQQQFQQDQRPQQQQQQLQKQQQQQGQSWRPQSHDPS
  Ago2.agam     (89) IRRKSKRSSRNREVHSSISRDLHNNSSNMRKNSMNRRSSNSSNHSAHSSS
   Ago2b.dm    (251) RPPGQQQGGHQQGRQGQEGGYQQRPPGQQQGGHEQGRQGQEGGYQQRPSG

                         301                                             350
   Ago2.aae    (129) PASGSHSHSSS--------------------------------P------
  Ago2.agam    (139) SSSNNSAHSSSNSSRAPHNSSSSNNSA---HGSSNTNRRFPLLPPGSAV-
   Ago2b.dm    (301) QQQGGHQQGRQGQEGGYQQRPSGQQQGGHQQGRQGQEGGYQQRPSGQQQG

                         351                                             400
   Ago2.aae    (141) -------------------SHAAALERVEEDFSKIKIDKQKIHSSALLPV
  Ago2.agam    (185) ------HRDASVASTKSSSSGDGTLQTIEENLGAMRIAKEKIRRTDLRPV
   Ago2b.dm    (351) GHQQGRQGQEGGYQQRPPGQQPNQTQSQGQYQSRGPPQQQQAAPLPLPPQ

                         401                                             450
   Ago2.aae    (172) LMRPNAHGTRGRAIKVEVNYIQLLLERLIPTAYHYDVDIQPAASRKWQRL
  Ago2.agam    (229) LVRRGAHGKRGKPVSVEANFFRLLLDKLKGTAYHYDVAIEPDRPKKFYRP
   Ago2b.dm    (401) PAGSIKRGTIGKPGQVGINYLDLDLSKMPSVAYHYDVKIMPERPKKFYRQ

                         451                                             500
   Ago2.aae    (222) AFSEFTKQMFPNHGFAFDGHKNAYAARRLQADVYEQEVKVRDE-GRERRF
  Ago2.agam    (279) VFAQFCRENYPGAMLAFDGQKNAYTTRKLSDKKAKVVFQPDDG-GKQREY
   Ago2b.dm    (451) AFEQFRVDQLGGAVLAYDGKASCYSVDKLPLNSQNPEVTVTDRNGRTLRY

                         501                                             550
   Ago2.aae    (271) KVAMKEAAVLDMTCLKTYMN-NGSTLDKPMSAIQCLDIVLRTAYENNPRF
```

```
Ago2.agam    (328) TVQVKEAAQLDLGVLKTYMKSNEETMAKPMSAIQCLDVVLRSAYENNPNF
 Ago2b.dm    (501) TIEIKETGDSTIDLKSLTTYMNDRIFDKPMRAMQCVEVVLASPCHNKAIR

                   551                                              600
 Ago2.aae    (320) IKFKKSIYVKPDRPDDIGSNHELWYGLFQSALLGARPFLNIDVSHKAFPT
Ago2.agam    (378) VRFKRAVYAVPRQSIDIGRGHELWFGLFQSALLGSRPYLNVDVSHKAFPM
 Ago2b.dm    (551) VGRSFFKMSDPNNRHELDDGYEALVGLYQAFMLGDRPFLNVDISHKSFPI

                   601                                              650
 Ago2.aae    (370) GGPVLRILVDMNRGQ--VPDRVTDWMSRDLHDFLKGMELSYTGPDG---V
Ago2.agam    (428) GAPVLKVIGDFNRG---QVDQVSGWVQQELHSFLKGMDVVYTNPTTR--M
 Ago2b.dm    (601) SMPMIEYLERFSLKAKINNTTNLDYSRRFLEPFLRGINVVYTPPQSFQSA

                   651                                              700
 Ago2.aae    (415) SKLFKYNSIKSPANQQKFKLENGTEMTIDQYFR-SKNKQLRYPSLPVLHV
Ago2.agam    (473) AKRMRCNGLREPASQQMFKLEDGTRLSVADYFARKLNFRLRYPNLPVLHV
 Ago2b.dm    (651) PRVYRVNGLSRAPASSETFEHDGKKVTIASYFH-SRNYPLKFPQLHCLNV

                   701                                              750
 Ago2.aae    (464) GSLVRNVMLPIELCSIPPGQALNKKHPDQCTQFIIRKSATDTATRKRKIM
Ago2.agam    (523) GSTVRSVYVPAELCDIPAGQALNKNNPEECTRDIIRYAATSAPERKRKIL
 Ago2b.dm    (700) GSSIKSILLPIELCSIEEGQALNRKDGATQVANMIKYAATSTNVRKRKIM

                   751                                              800
 Ago2.aae    (514) DLFNQIGYNNAPTIKEFGVSVGNNFETVDGRILDPPELSYRNDRRVKPMR
Ago2.agam    (573) DLASQIQYNKCPTLLDFGITVGNEFEKVPARIIDAPPIEYARGEKIPPQR
 Ago2b.dm    (750) NLLQYFQHNLDPTISRFGIRIANDFIVVSTRVLSPPQVEYHSKRFTMVKN

                   801                                              850
 Ago2.aae    (564) GVWRADNMNFIIPSTEITRRELSWTILNLDGRTRPDAIDEFGRNIYQMSL
Ago2.agam    (623) GVWRAEGKNFIVPSTELSKRPLRWRILNLDSYTNEATVKKFGEMLQSQAM
 Ago2b.dm    (800) GSWRMDGMKFLEPKPKAHKCAVLYCDPRSGRKMNYTQLNDFGNLIISQGK

                   851                                              900
 Ago2.aae    (614) KQGVQLQQFSMKNNFYEPRDMRFAVKDLDNIFDELKKRKIDLVFVVIPSP
Ago2.agam    (673) RCNVQMEPFDMAKTYVLVRDMRNCLRDIGTLLQNIKREEPAVTIVVLPSR
 Ago2b.dm    (850) AVNISLDSDVTYRPFTDDERS------LDTIFADLKRSQHDLAIVIIPQF

                   901                                              950
 Ago2.aae    (664) GRDGDVYAKVKQKAELC---VGLLTQCIKSFTLDKKRGDMSTISNIWLKI
Ago2.agam    (723) ---GDAYAKVKQKAELASERIGLLTQCVKGMTVAKKGTDMSTLNNIMLKI
 Ago2b.dm    (894) R--ISYDTIKQKAELQH---G-ILTQCIKQFTVER-KCNNQTIGNILLKI

                   951                                             1000
 Ago2.aae    (711) NAKTNGSNHVLAKNFKPPIARKTVMYVGADVTHPSPEQTNIPSVVGVAAS
Ago2.agam    (770) NAKTNGTNHCISQVAVPPLGRGKVMYIGADVTHPLSE--NEPSVVGVAAL
 Ago2b.dm    (937) NSKLNGINHKIKDDPRLPMMKN-TMYIGADVTHPSPDQREIPSVVGVAAS

                   1001                                            1050
 Ago2.aae    (761) YDLEGFRYNCCYRLQGPKDEMIRDLQNIVIKQLRQFKQTNQSLPELIMYY
Ago2.agam    (818) YDLTGFRYNCSVRLQGARDEMIRDLENIVQRQLLLYKQYNGALPERIMYY
 Ago2b.dm    (986) HDPYGASYNMQYRLQRGALEEIEDMFSITLEHLRVYKEYRNAYPDHIIYY

                   1051                                            1100
 Ago2.aae    (811) RDGVSEGQFQEVLTIELRAMQAAAASVQQGYKPNITFIVVQKRHHARFFP
Ago2.agam    (868) RDGVSDGQFAEILTIELQALHAAIARVEPGYKPAVTFIVVQKRHHTRFFP
 Ago2b.dm   (1036) RDGVSDGQFPKIKNEELRCIKQACDKVG--CKPKICCVIVVKRHHTRFFP
```

```
                           1101                                          1150
   Ago2.aae    (861)  TANCPTEGRNNNVQPGTIVDRYITAPNQYQFFLVSHAAVQGVAKPTKYCV
  Ago2.agam    (918)  QPGCPTEGKNGNVPPGTIVDSEITTPDRYEFYLVSHAAVQGVAKPTKYVV
   Ago2b.dm   (1084)  SGDVTTSNKFNNVDPGTVVDRTIVHPNEMQFFMVSHQAIQGTAKPTRYNV

                           1151                                          1200
   Ago2.aae    (911)  LYDDENCNPDQLQALTYYLCHMFTRCNRAVSYPAPTYYAHLAAYRGRVYI
  Ago2.agam    (968)  LYDDSNCHPDSLQALTYNLCHLFARCNRAVSYPAPTYYAHLAAYRGRVYI
   Ago2b.dm   (1134)  IENTGNLDIDLLQQLTYNLCHMFPRCNRSVSYPAPAYLAHLVAARGRVYL

                           1201                          1232
   Ago2.aae    (961)  KDRPLNMNNLTKEYERMQIRTEIQDGHPMFFV
  Ago2.agam   (1018)  KDRRINMNDMENAYRDIQIIDTVNNSTPMFFV
   Ago2b.dm   (1184)  TG-TNRFLDLKKEYAKRTIVPEFMKKNPMYFV
```

Additional file 1B

```
                           1                                               50
   Dcr2.aae      (1)  MDMIMPQQDDFIPRDYQRTMKTICMQKNTIIYLPTGAGKTHIALMVIKEM
   Dcr2agam      (1)  -SQNKEPMEDFAPRNYQVQMKEICLAKNTIIFLPTGSGKTYIALMVMKEI
    Dcr2.dm      (1)  -----MEDVEIKPRGYQLRLVDHLTKSNGIVYLPTGSGKTFVAILVLKRF

                           51                                             100
   Dcr2.aae     (51)  GKDLDKPLTEGGKRTFFVVNTVALAKQQAEFLSHNLTYDTSIYTSDRNVD
   Dcr2agam     (50)  SHQLRNTVHEGGKRTFFLANTVALAKQQAQFFARHMPFNVRLYTSEVNVD
    Dcr2.dm     (46)  SQDFDKPIESGGKRALFMCNTVELARQQAMAVRRCTNFKVGFYVGEQGVD

                           101                                            150
   Dcr2.aae    (101)  AWKQDKWLEEFAKYQVIVCTCQILLDVLKHGYLSVKHINLLIFDECHHGV
   Dcr2agam    (100)  AWKSDRWHEEFSEGQVIICTAQILLDVLRHGYMSPANINLIVFDECHRAV
    Dcr2.dm     (96)  DWTRGMWSDEIKKNQVLVGTAQVFLDMVTQTYVALSSLSVVIIDECHHGT

                           151                                            200
   Dcr2.aae    (151)  GEHPMHGIMEQFLRVPKSDHPRVIGLSGMLLYKQIKSVALVSPELERLEN
   Dcr2agam    (150)  GQHPMHAIMKEIVAAPASERPRVLGLSGTLLFKELKMASQVPDELERLEN
    Dcr2.dm    (146)  GHHPFREFMRLFTIANQTKLPRVVGLTGVLIK--GNEITNVATKLKELEI

                           201                                            250
   Dcr2.aae    (201)  TFNATIATVGSYDAFTEVCKFSTDPNELLVSYST--LRLSPVMADIVNNI
   Dcr2agam    (200)  TFSSTIATVANYDDYATVASFSTNPNEVLVTYSKPAVHLMPLVKEMWPRI
    Dcr2.dm    (194)  TYRGNIITVSDTKEMENVMLYATKPTEVMVSFPH-QEQVLTVTRLISAEI

                           251                                            300
   Dcr2.aae    (249)  NAFSQTIEEFHLPKYLNQ-NKALLKDRP-KPLKEIRKLFTELIYQLGDTG
   Dcr2agam    (250)  DAFVEWLLQVYLPEYSTQSTRTLQKSFC-KPLKEVKRALTEFKHQLEEHG
    Dcr2.dm    (243)  EKFYVSLDLMNIGVQPIRRSKSLQCLRDPSKKSFVKQLFNDFLYQMKEYG

                           301                                            350
   Dcr2.aae    (297)  LFGGSIALLGLIVQFELDKRQSDSSMLRLALRSCITFCESLRHQIEKLMS
   Dcr2agam    (299)  MYAGSLAILAVIVQLEVSKRQSPCDKARQVYRSAISFCESIRHELVEAMS
    Dcr2.dm    (293)  IYAASIAIISLIVEFDIKRRQAETLSVKLMHRTALTLCEKIRHLLVQKLQ

                           351                                            400
```

```
Dcr2.aae  (347) ---------GLDMKTKLTKFSSLKVRQLIDQLEKLYEENRDKKAKTLIFV
Dcr2agam  (349) ---------GLRGTHQILSFSSDQARKLLKYLEDSYRTAEDKNKQALVFV
 Dcr2.dm  (343) DMTYDDDDDNVNTEEVIMNFSTPKVQRFLMSLKVSFADKDPKDICCLVFV

                401                                             450
Dcr2.aae  (388) QRRFSAKVLYHLLKIYFAETEDANLIVPDFMVGNNGSMPESIEQILSAKK
Dcr2agam  (390) KRRFTAKVLYHLIRIYFHYELVEPIVKPDFIVGANAALEESIDAILVVRE
 Dcr2.dm  (393) ERRYTCKCIYGLLLNYIQSTPELRNVLTPQFMVGRNNISPDFESVLERKW

                451                                             500
Dcr2.aae  (438) DRRVLERFKKNETNVIVTTNVLEEGIDLQMCNTVVKYDHPQTFASYQQSK
Dcr2agam  (440) DRRVIENFRKRKINVLCATNVLEEGIDLQMCNMVIMYDAPLSYASFMQSK
 Dcr2.dm  (443) QKSAIQQFRDGNANLMICSSVLEEGIDVQACNHVFILDPVKTFNMYVQSK

                501                                             550
Dcr2.aae  (488) GRARMKNSQYMVMLDNENRHIFLEKYRLYKSIEEELRRCLIGKTINRPDP
Dcr2agam  (490) GRARMKTSTYLMMTPAADLQQFAKRMKLYRDIENRLKEELVGKTINRPEP
 Dcr2.dm  (493) GRARTTEAKFVLFTADKEREKTIQQIYQYRKAHNDIAEYLKDRVLEKTEP

                551                                             600
Dcr2.aae  (538) LDADVHKELYNEIIPPFFTAKGAKLDALSAIQLLNRYCMGMPRDAFTNTN
Dcr2agam  (540) LENDVRKELLDDLIPPFYTPFKAKLDALSAIQLLNRYCMSMPRDLFTGSN
 Dcr2.dm  (543) ELYEIKGHFQDDIDP-FTNENGAVLLPNNALAILHRYCQTIPTDAFGFVI

                601                                             650
Dcr2.aae  (588) VTWERIDLKD----------GRIIVEVLLPLQSTVREKISGNPMRNIKLA
Dcr2agam  (590) VTWERIDRSP----------TEIIVTVKLPLQSTVREVIHGQTMKNLKLA
 Dcr2.dm  (592) PWFHVLQEDERDRIFGVSAKGKHVISINMPVNCMLRDTIYSDPMDNVKTA

                651                                             700
Dcr2.aae  (628) KRSAAFNACRKLYENKELNEHLIPIDCKYQLNNLKDVYFRHWKDFDADLG
Dcr2agam  (630) KQSAAFNACKRLFEVGELNMYLLPIATKDKVEELSEQYFKLWRKMSDEPN
 Dcr2.dm  (642) KISAAFKACKVLYSLGELNERFVPKTLKERVASIADVHFEHWNKYGDSVT

                701                                             750
Dcr2.aae  (678) --KLAGTQKCIRTHAIQYPKQTTECFPQPG-KPCYIYVLRIAAGFAQDPT
Dcr2agam  (680) P-KQAGTMKYVRGHKIVYPEETVGCTPQADGEQCYVYIVRMRAHFDANTH
 Dcr2.dm  (692) ATVNKADKSKDRTYKTECPLEFYDALPRVG-EICYAYEIFLEPQFESCEY

                751                                             800
Dcr2.aae  (725) NDNVNIFHSLYSSENNFGLMTTKPLPALAKMKFFVTLGLINVHIEETPIV
Dcr2agam  (729) LENVRIFQELYSSANNFGILTRKRLPRLARMKLFVTLGAIGVEIVPEPVC
 Dcr2.dm  (741) TEHMYLNLQTP---RNYAILLRNKLPRLAEMPLFSNQGKLHVRVANAPLE

                801                                             850
Dcr2.aae  (775) LPNGGSEIELALLRQFHVTVFRDVLKLWKEFLCCDYDNEENSFLVVPLKN
Dcr2agam  (779) ITLAPDSGELQRLKRFHLLLFRDLLKVWKPFTVLDALPEENGFLIVPMLR
 Dcr2.dm  (788) VIIQ-NSEQLELLHQFHGMVFRDILKIWHPFFVLDRRSKENSYLVVPLIL

                851                                             900
Dcr2.aae  (825) STH----LDWKLIREFQNLSEPPSEISTIARNKMEFEADKYRHKVILPWY
Dcr2agam  (829) SQS----IDWELMGKFPYLR-PAAETSTRARQHLRFETEQYLLRVVHPWY
 Dcr2.dm  (837) GAGEQKCFDWELMTNFRRLPQSHGSNVQQREQQPAPRPEDFEGKIVTQWY

                901                                             950
Dcr2.aae  (871) KNNKEQPYVVTMVHEHLTPESPFPNPEYGSYANYFSQAYHLAVVK-PDQF
Dcr2agam  (874) KNDPDQNYVVVRVRDDLRPTSPFPNAKYDSYEQYFAQEHHQVVVRHEDQF
```

```
Dcr2.dm    (887) AN-YDKPMLVTKVHRELTPLSYMEKNQQDKTYYEFTMSKYGNRIGDVVHK

                 951                                              1000
Dcr2.aae   (920) LIEVKGITSYLNRLNPGVEDDG-KSTRSKHWRFNEILIPELCHNYQFPAD
Dcr2agam   (924) LIEVKGITTSLNRLHPGAEADGGASTRSRYWEFQEILIPELVHNFEFPAD
Dcr2.dm    (936) DKFMIEVRDLTEQLTFYVHNRG-KFNAKSKAKMKVILIPELCFNFNFPGD

                 1001                                             1050
Dcr2.aae   (969) YWLKATLLPSALHRLHYLLLAENIRVDLATGANVGCLENHTIEDVDVEYK
Dcr2agam   (974) YWLKATLLPSALHRVHYLLLAEGIRVDLARNAQVGSEHCERVEDVIIDRV
Dcr2.dm    (985) LWLKLIFLPSILNRMYFLLHAEALRKRFNTYLNLHLLPFNGTDYMPRPLE

                 1051                                             1100
Dcr2.aae  (1019) ERKGKQLEELQLMEFEEDEDEDDEFDLEEAKRSLVAPEN------LSELA
Dcr2agam  (1024) LSIEHQKSTALYGGEDDDDDEEDEDEDEEEKDSDEEGARSMQLLQVLNQI
Dcr2.dm   (1035) IDYSLKRNVDPLGNVIPTEDIEEPKSLLEPMPTKSIEAS--------VAN

                 1101                                             1150
Dcr2.aae  (1063) RNQMCSITGDIPLPWQEDEEPVDIERNWDQVSKLDLDYYNVFVNKFSDLS
Dcr2agam  (1074) HSDSISLADRVQYPWEENEQPKDLERNWDTVSKIDIDYYASFVKKYEQQA
Dcr2.dm   (1077) LEIT-----EFENPWQKYMEPVDLSRNLLSTYPVELDYYYHFSVGNVCEM

                 1151                                             1200
Dcr2.aae  (1113) MREKAAERISTAYTSAVYRR----------AAGSPKREPMAILDVPVDQK
Dcr2agam  (1124) VTITNIDRMLANLNMNKNE------------AEASPVKALPALEDGHGPV
Dcr2.dm   (1122) NEMDFEDKEYWAKNQFHMPTGNIYGNRTPAKTNANVPALMPSKPTVRGKV

                 1201                                             1250
Dcr2.aae  (1153) FAIKLLQLTPANTVNVNLQQKNIIKALTTKSSSDVFDLERYELLGDAFLK
Dcr2agam  (1162) AQIAMLRLTMDNTANVALQQSDLLQALTTKSSADVFNLERFEVLGDAFLK
Dcr2.dm   (1172) KPLLILQKTVSKEHITPAEQGEFLAAITASSAADVFDMERLEILGDSFLK

                 1251                                             1300
Dcr2.aae  (1203) FSISLYLVKYHKEWHEGFLTAVKGQIVSNRNLVYCAIKYGLPGMLKIHKF
Dcr2agam  (1212) FAVSVYILFRHTSWHEGYLTTCKGRMVSNRNLLYCAMGYGLPGKIKAHPF
Dcr2.dm   (1222) LSATLYLASKYSDWNEGTLTEVKSKLVSNRNLLFCLIDADIPKTLNTIQF

                 1301                                             1350
Dcr2.aae  (1253) DPKNDWQPPLATVPKNIKRTMQSVN------HSARVLYRLTLTEEEIKTG
Dcr2agam  (1262) DPKNDWVPPLSTVPGAVRRAMVDAN------ESPALLYNLKLTEEEIQSG
Dcr2.dm   (1272) TPRYTWLPPGISLPHNVLALWRENPEFAKIIGPHNLRDLALGDEESLVKG

                 1351                                             1400
Dcr2.aae  (1297) VVTAKNSDDFIAQLELHGN-----MPDPSPMANYLSQQTMGDKTPADAME
Dcr2agam  (1306) VVAQATVDKFLPLIEQAP------APSQSTLHTVLQQVQIRDKVVADVTE
Dcr2.dm   (1322) NCSDINYNRFVEGCRANGQSFYAGADFSSEVNFCVGLVTIPNKVIADTLE

                 1401                                             1450
Dcr2.aae  (1342) ALLGVCVQSVGIERSFKLLPHFGILPKTHNVLRLLADKIENQ--RLKTHI
Dcr2agam  (1350) ALLGVCVKTVGYERSFRFLSHLGIIPKGADVPMLLRTTTFP---IGDYFP
Dcr2.dm   (1372) ALLGVIVKNYGLQHAFKMLEYFKICRADIDKPLTQLLNLELGGKKMRANV

                 1451                                             1500
Dcr2.aae  (1390) DIREVDAFLKNYRRIEGILGYKFKDRTYLLQALTHASYPTNRITGSYQQL
Dcr2agam  (1397) VRHKVDQLLCNPERIEATLGYRFRNRTYLLQAFTHTSYTSNSLTGSYQQL
Dcr2.dm   (1422) NTTEIDGFLINHYYLEKNLGYTFKDRRYLLQALTHPSYPTNRITGSYQEL
```

```
                 1501                                             1550
Dcr2.aae  (1440) EFLGDAVLDFLISMYIFEQNPTMSPGQLTDLRSALVNNVTLACILVRHGL
Dcr2agam  (1447) EFLGDAVLDFLVSMYIYERNPSMSPGQLTDLRSALVNNVNLACVLVRNEL
 Dcr2.dm  (1472) EFIGDAILDFLISAYIFENNTKMNPGALTDLRSALVNNTTLACICVRHRL

                 1551                                             1600
Dcr2.aae  (1490) HLYILAESASFTDTVSKFVLFQEQHKHEITDQVNLLVEESDRK-------
Dcr2agam  (1497) HRHILSQSPMLTDAIGKFVAVHRCHRNQGSNWVRLLTEESDTP-------
 Dcr2.dm  (1522) HFFILAENAKLSEIISKFVNFQESQGHRVTNYVRILLEEADVQPTPLDLD

                 1601                                             1650
Dcr2.aae  (1533) ------------------------------MAEFVDVPKALGDVFESLVA
Dcr2agam  (1540) ------------------------------MAEYVDVPKVLGDVLEALIG
 Dcr2.dm  (1572) DELDMTELPHANKCISQEAEKGVPPKGEFNMSTNVDVPKALGDVLEALIA

                 1651                                             1700
Dcr2.aae  (1553) AVFLDSGNDFAATWQVIYGMMGNEILTFTENTPIQIVRQLYEFKPSCKPT
Dcr2agam  (1560) AIYLDSGNDLAATWEVCFRLLRDEIADFTRKTPIQVVRQLYEHP-EASPH
 Dcr2.dm  (1622) AVYLDCR-DLQRTWEVIFNLFEPELQEFTRKVPINHIRQLVEHK-HAKPV

                 1701                                             1750
Dcr2.aae  (1603) FSRAIPDEDTVLVKLRYEIRNQQHEAYGFGQNKDDAKRAAAKAALQVLRK
Dcr2agam  (1609) FSAPFVEEEVVYVKLSYTHRSQRQTVYGFGKNKDDAKRAAAKIALSKIM-
 Dcr2.dm  (1670) FSSPIVEGETVMVSCQFTCMEKTIKVYGFGSNKDQAKLSAAKHALQQLSK

                 1751
Dcr2.aae  (1653) HYRSAK-
Dcr2agam  (1658) -------
 Dcr2.dm  (1720) CDA----
```

Additional file 1C

```
               1                                                50
 TSN.aae   (1) --------MSAAPAASAPAPAPVLKRGIVKQVLSGDSVIIRGQPKGGPPP
TSN.agam   (1) -----MSAANVPAAAANPAPPPVLKKGIVK-ILSGDSLILRDKPVNGPPR
  TSN.dm   (1) MATAANTATAAGAAKDAPPAPTKSLSGIVKQVLSGDTVVIR-ATKGAPPP

               51                                              100
 TSN.aae  (43) EKQINFSGVIAPKLARRPTNNSTEITKDEPYAWEAREYLRQRLIGQEVYF
TSN.agam  (45) EKQLNFAGIVAPKLARRPTNGSSDGSRDQPYAWESREYLRQRLIGQEVWF
  TSN.dm  (50) EKQITFSHVLAPKLARRPG-AGGDETKDEPWAWESREFLRKKLIGVEVTF

               101                                             150
 TSN.aae  (93) YSERPPNATRDYGYVCLGKDPATSENIVESIVSEGLVSVRREGVRQTPEL
TSN.agam  (95) YSEKPPNANREYGYVKLGKEPN-AENIVESIVSEGLVTVRRDNVRQTPEH
  TSN.dm  (99) TFDKPANSNREYGFVWIGKDKETGENVVESIVREGLVSVRRE-GRPTAEQ

               151                                             200
 TSN.aae (143) TRLCELEDAAKAARKGKWSDSP-SSDHVRNITWNIENPKAFFDHHNGKPI
TSN.agam (144) ARLIELEDAARRARKGLWSDAP-EGEHVRNIVWNIDNPKQFVDQHAGQLI
  TSN.dm (148) QTLIELEDQARAAGRGKWSPTASAADKVRNIKWSHENPAHLVDIYGGNPV

               201                                             250
 TSN.aae (192) KAIIEHVRDGSTVRAFLLP---EFQHVTLMMSGIRCPGFKLDVDGKPDTT
```

```
TSN.agam   (193) KAIIEHVRDGSTVRAFLMPNPRVFQHVTLMMSGIRCPGFKLDAEGRPDNT
  TSN.dm   (198) KAIIEHVRDGSTVRAFLLP---DFHYITLMISGIRCPGVKLDADGKPDLS

                 251                                            300
 TSN.aae   (239) AEVPFAEEARYFVESRLLQRDVEIRLESVNNSNFVGTIIFPKGNIAEALL
TSN.agam   (243) TEVPYADEARFHVECRLLQREVKVRLESNSNTNFLGTILCPEGNIAESLL
  TSN.dm   (245) VKVPFADEARYYVETRLLQRDVEIRLESVNNSNFIGTILYPKGNIAESLL

                 301                                            350
 TSN.aae   (289) KEGFAKCVEWSMPYVKEGVDRLRAAEKHAKGNRLRLWKDYQAPTAAYNTK
TSN.agam   (293) RNGFAKCVEWSIPYVKEGIDRLRACEREAKAARLRLWKDYKPPAALANTK
  TSN.dm   (295) REGLAKCVDWSMAVMKTGTDKLRAAERFAKEKRLRQWQDYQAKTPAFNSK

                 351                                            400
 TSN.aae   (339) DKDFTGTVVEVFNGDAVMVKISNTVSKKVFLSSIKPPREA----ARTADE
TSN.agam   (343) DKELVGTVMEVYNGDAVLVKVG-TVSKKVFFSSIRPP--------RPKED
  TSN.dm   (345) EKDFSGTVVEVFNGDAINVRLSNGQVKKVFFSSIRPPRDQRAVVGTDGEE

                 401                                            450
 TSN.aae   (385) EGNLPPRPKGSRPLYDVPWMFEAREFLRKKLIGKKVHCSLDYVTPARDNF
TSN.agam   (384) DG---PRAKNSRPLYDIPYMFEAREFLRKKLIGKRVTCTLDYVAPARDNY
  TSN.dm   (395) IVKAPPRGKNYRPLYEIPHMFDAREFLRKKLINKKVQCNLDYISPPRENF

                 451                                            500
 TSN.aae   (435) PEKCCYTVTLSGANVAEALVAKGLATVIKYRQDDDQRSVHYDELRSAETQ
TSN.agam   (431) PEKYCYTVRLDDQNIAEAMLERGLATVINYRQDDEQRSPEYDKLRAAQEQ
  TSN.dm   (445) PEKYCYTVSIGGQNVAEAMVAKGLATCVRYRQDDDQRSSAYDQLIAAEQQ

                 501                                            550
 TSN.aae   (485) AMKQLKGVHAKDDIPSHRINDLTVDHSRIKHQYLPSWQRALRTEAIVEFV
TSN.agam   (481) AIKGQKGMHAKKQTPSHRINDLTTDHSRIKHHYLPSWQRALRTEALVEFV
  TSN.dm   (495) AIKGLKGLHAKKDNATLRVNDLTVDHSRIKVQYLPSWQRALRTEAIVEFV

                 551                                            600
 TSN.aae   (535) ASGSRFRIYCPKDSCLVTFLLAGISCPRSSRPALSGVPAQEGEPFGDEAL
TSN.agam   (531) ASGSRLRLYCPKESCLVTFLLAGISCRRSSRPAIGGAPAQEAEPYGDEAL
  TSN.dm   (545) ASGSRLRIFVPKDSCLVTFLLAGISCPRSSRPALNGVPAQEGEPFGDEAL

                 601                                            650
 TSN.aae   (585) QFSKERILQRDVSVKIETTDKAATSVIGWLWTENNVNLSVALVEEGLASV
TSN.agam   (581) QFTREKVLQRDVSVKIETTDKQATSVIGWLFTDHNVNLSVALVEEGLAEV
  TSN.dm   (595) TFTRERVLQRDVSVHIDTTDKAGSSVIGWLWTDSGANLSVALVEEGLAEV

                 651                                            700
 TSN.aae   (635) HFTAEKTEHFRALSEAEARAKAKRKNIWKDYVEKVEEDN-KENEDEKDDP
TSN.agam   (631) HFTAEKSDYYRVLRDAEARAKAQRKNIWKDYVEKAAAEEEKDEIEDTPDV
  TSN.dm   (645) HFSAEKSEYYRQLKIAEDRAKAAKKNIWTNYVEEVPKEK--TVTEEEKED

                 701                                            750
 TSN.aae   (684) AAPADRKVKYENVVVTEVTPELHFYAQHADQGAKLEELMTKLRQEFRAMP
TSN.agam   (681) NTPVERKVKYESVVVTEVTPELQFYAQHTDQGAKLEELMTKLRQDFKAMP
  TSN.dm   (693) KVVAERKVNYENVIVTEITETLTFFAQSVESGSKLESLMSKLHADFQSNP

                 751                                            800
 TSN.aae   (734) PVTGAYNPRRGDMCAAKFSEDNEWYRAKVEKIEKGGNASILYVDYGNRET
TSN.agam   (731) PVTGSYAPKRGDMCAAKFSEDNEWYRAKVEKVEKGGNVTILYIDYGNRET
  TSN.dm   (743) PIAGSYTPKRGDLVAAQFTLDNQWYRAKVERVQ-GSNATVLYIDYGNKET
```

```
                  801                                               850
 TSN.aae   (784)  VPTTRLAMLPPAFISDKPYAHEYSLALVVLPTDEEDKADALKAFAQDALN
TSN.agam   (781)  VPSTRLAMIPPTFISEKPFAHLYVPALLLLPTDADDRAEAVKAFSQDVLN
  TSN.dm   (792)  LPTNRLAALPPAFSSEKPYATEYALALVALPTDNEDKEEALRAFSEDVLN

                  851                                               900
 TSN.aae   (834)  KTLQMNVEYRVSG-AEHVTLVDPATKVDIGKELVSDGFLIAEKNKKDRRL
TSN.agam   (831)  RTLNMNVEYRISG-TEYVTLTDPATKADIAEDLIADGYLIADKNKKDRRL
  TSN.dm   (842)  HKVQLNVELKVTGSPNLATLRDPTTKVDFGKQLVAEGLVLAEQ-RGERKL

                  901                                 939
 TSN.aae   (883)  QKLINDYKEAEQSARKNRNGIWQYGDSTEDQAGEFGLSR
TSN.agam   (880)  TKLIADYKDAE----------------------------
  TSN.dm   (891)  KELVDQYKAAQEAARVAHLAIWKYGDITQDDAPEFR---
```

Additional file 1. Amino acid conservation of Ago2, Dcr2, and TSN across species. (A) Ago2. (B) Dcr2. (C) TSN. Black shaded sequence indicates a.a. identity; gray shaded sequence indicates a.a. similarity. *Ae. aegypti*, Ago2. aae, [Vectorbase: SUPP_AEDES003395]; *An. gambiae*, Ago2.agam, [Genbank: XM_559969]; *D. melanogaster*, Ago2.dm isoform B, [Genbank: NP_648775]. Dcr2.aae, [Genbank: AY713296]; Dcr2.agam, [Genbank: XM_320248]; Dcr2.dm, [Genbank: NM_079054]. TSN.aae, Genbank: [AAEL000293], Genbank: [NP_612021], Genbank: [XM_315689].
