# Supplementary material for: Aedes aegypti uses RNA interference in defense against Sindbis virus infection
Source: BMC Microbiol. 2008 Mar 17;8:47. doi: 10.1186/1471-2180-8-47 (PMC2278134; doi:10.1186/1471-2180-8-47)
Supplement: Additional file 3 — Dcr2 silencing does not increase mosquito mortality during MRE16-eGFP infection. Life Table analysis. [file 1471-2180-8-47-S3.pdf]

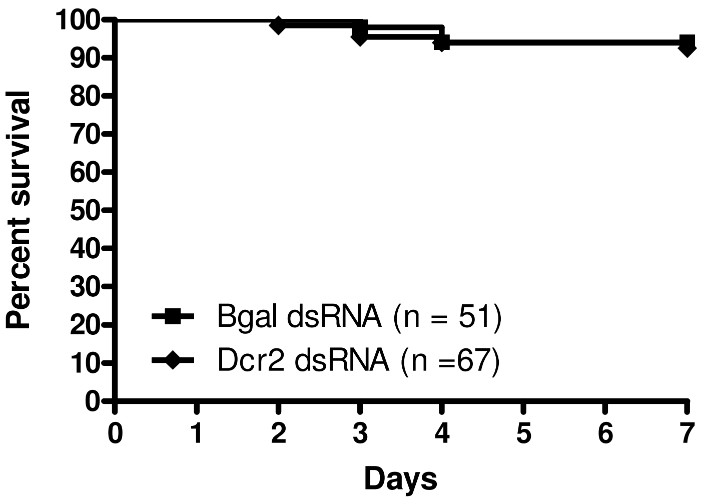

Additional File 3. Dcr2 silencing does not increase mosquito mortality during MRE16-eGFP infection. Mosquitoes were injected with dsRNA as indicated and held for three days prior to virus feed; survival was monitored daily.
