# Supplementary material for: Aedes aegypti uses RNA interference in defense against Sindbis virus infection
Source: BMC Microbiol. 2008 Mar 17;8:47. doi: 10.1186/1471-2180-8-47 (PMC2278134; doi:10.1186/1471-2180-8-47)
Supplement: Additional file 4 — Primer Table. PCR primer sequences. [file 1471-2180-8-47-S4.pdf]

Campbell and others, BMC Microbiology

Additional File 4 Primers

	target	Forward Primer	Reverse primer
qRT-PCR	*ACT1*	nt301- GAGCACCCAGTTCTGCTGAC	nt446- GTACGACCGGAGGCGTACAG
	TSN	nt2631- CACGACCCGCCTAGCCATGCTT	nt2682-GTACGGTTTGTCCGAAATGAAG
	Dcr2(3)	nt4546- GAATTCCTCGGCGATGCGGTATTA	nt4724- GATGCCGACTCTGCCAGGATGT
	Ago2	nt2558- GACACCATGCCCGTTTCTTC	nt2680- CCAGGAAGAATTGATACTGGTT
	Ago2(4)	nt2045- GGCTGCTCACCCAATGTATCAAGA	nt2149- AACCGTTCGTTTTGGCGTTGAT
	TR339	nt4495- TAGACAGAACTGACGCGGACGT	nt4635-TCCATACTAACTCATCGTCGATCTC
	MRE-16	nt4494- TGGACAGAACTGACGCAGAC	nt4699- CGAGTAGAGTTTGCCCTTCG
dsRNA	*Ago2* *	nt2383- CAGTTCAAGCAGACGAACCA	nt2881- TGATGTAGACGCGTCCTCTG
	*Dcr2**	nt4006-CCACCTCTAGCAACGGTTCCGAAGA	nt4504- CGCATGGGTGAGTGCTTGTAGGAG
	*TSN**	nt2713-CTGCCAACCGACGAAGAGGACA	nt3089- TGAAGGGCAGGTGTGAACATCG
	TR339-nsP3*	nt4549- AAAGAATCGACGCGGCAC	nt5032- AGGACTACTTTCGTGCACTG
	β-GAL*	GGTCGCCAGCGGCACCGCGCCTTC	GCCGGTAGCCAGCGCGGATCATCGG

Probe	MRE16-eGFP	* nt9976- TATGATCGTCTGAAGGAAACATCAG	nt12182- GCA AAC AGC CAG CTC CAT GAT G
		*nt4976- ACTTCCGAAGCACAAAATTAAGAAC	nt9083- GGGTGTTCGCTGGTGTAGGAGA
		*nt1- GATTGGCGGCGTAGTACACACTAT	nt4899- ACATACACAAACAGGGAAGCGTCT
		nt9976- TATGATCGTCTGAAGGAAACATCAG	*nt12182- GCA AACAGCCAGCTCCATGATG
		nt1- GATTGGCGGCGTAGTACACACTAT	*nt5000- GTTCTTAATTTTGTGCTTCGGAAGT
		nt4976- ACTTCCGAAGCACAAAATTAAGAAC	*nt10000- CTGATGTTTCCTTCAGACGATCATA

Additional File 4. Primers used for qRT-PCR and dsRNA injections.
Primer nucleotide (nt) position numbers correspond to the published sequence: *Act1*, [Genbank: AAU20287]; *TSN*, [Genbank: AAEL000293]; *Dcr2*, [Genbank: AY713296]; *Ago2*, [Genbank: SUPP_AEDES003395]; TR339, [Genbank: NC_001547]; MRE16, [Genbank: AF492770]. *Ago2* primers were used to analyze transcript levels. *Ago2(4)* primers were used to analyze transcript levels following Ago2 dsRNA injection. "*" indicates placement of 5' T7 promoter extension on dsRNA and viral probe primers (TAATACGACTCACTATAGG).
